# Impact of multidrug resistance on the virulence and fitness of *Pseudomonas aeruginosa*: a microbiological and clinical perspective

**DOI:** 10.1007/s15010-024-02313-x

**Published:** 2024-07-02

**Authors:** Elena Sendra, Almudena Fernández-Muñoz, Laura Zamorano, Antonio Oliver, Juan Pablo Horcajada, Carlos Juan, Silvia Gómez-Zorrilla

**Affiliations:** 1 Infectious Diseases Service, Hospital del Mar, Infectious Pathology and Antimicrobials Research Group (IPAR), Hospital del Mar Research Institute, Universitat Autònoma de Barcelona (UAB), CEXS-Universitat Pompeu Fabra, Passeig Marítim 25-27, 08003 Barcelona, Spain; 2 Research Unit, University Hospital Son Espases-Health Research Institute of the Balearic Islands (IdISBa), Microbiology Department, University Hospital Son Espases, Crtra. Valldemossa 79, 07010 Palma, Spain; 3 Center for Biomedical Research in Infectious Diseases Network (CIBERINFEC), Instituto de Salud Carlos III, Madrid, Spain

**Keywords:** *Pseudomonas aeruginosa*, Multidrug resistance, Virulence, Fitness, Biological cost, Difficult to treat resistance, Antibiotic resistance

## Abstract

*Pseudomonas aeruginosa* is one of the most common nosocomial pathogens and part of the top emergent species associated with antimicrobial resistance that has become one of the greatest threat to public health in the twenty-first century. This bacterium is provided with a wide set of virulence factors that contribute to pathogenesis in acute and chronic infections. This review aims to summarize the impact of multidrug resistance on the virulence and fitness of *P. aeruginosa*. Although it is generally assumed that acquisition of resistant determinants is associated with a fitness cost, several studies support that resistance mutations may not be associated with a decrease in virulence and/or that certain compensatory mutations may allow multidrug resistance strains to recover their initial fitness. We discuss the interplay between resistance profiles and virulence from a microbiological perspective but also the clinical consequences in outcomes and the economic impact.

## Introduction

*Pseudomonas aeruginosa* is one of the top nosocomial pathogens and thus a leading cause of severe hospital-acquired infections, particularly in critically ill and immunocompromised patients [1]. As a proof of its clinical relevance, for instance *P. aeruginosa* is responsible for approximately 4.7–8.9% of hospital-acquired bloodstream infections and multidrug resistant (MDR) *P. aeruginosa* responsible of 26.3% of MDR bacteremia [2–4],with mortality rates of around 30% [5–7].

Different studies have shown that *P. aeruginosa* infections are associated with worse clinical outcomes than other pathogens [8–15]. To give just one example, Thaden et al. evaluated the impact of bacterial species (*Staphylococcus aureus* or gram-negative bacteria) on outcome in bloodstream infections in a 13-year prospective study involving more than 2,600 hospitalized patients with bloodstream infection and found a significant association between *P. aeruginosa* and higher in-hospital mortality compared to other bacteria after adjusting for patient demographics, medical comorbidities, bacterial antibiotic resistance, and treatment factors [16].

A key aspect of the success of *P. aeruginosa* as an opportunistic pathogen is its extensive genomic complexity and metabolic plasticity, which includes several intrinsic defense mechanisms, an extensive array of virulence genes and biofilm production capacity [17]. Connected to this is the extraordinary capacity of this species to develop and spread antimicrobial resistance in vivo [18, 19].

The worldwide emergence of antimicrobial resistance has become a major public health concern [20]. *P. aeruginosa* is one of the top six pathogens responsible for resistance-associated deaths [20] and is listed among the ESKAPE pathogens [21]. The WHO lists carbapenem-resistant (CR) *P. aeruginosa* among the ‘critical priority’ pathogens for which new antibiotics are urgently needed [22].

Regarding *P. aeruginosa* epidemiology, in a recent study conducted from 2018 to 2022 by the ATLAS (Antimicrobial Testing Leadership and Surveillance) global program, the rates of carbapenem-resistant *P. aeruginosa* varied from 15 to 33% depending on the region. Difficult-to-Treat Resistant *P. aeruginosa* rates varied from 6% in North America to 12% in Latin America in 2022 and remained stable over time in all global regions, except for a signifcant decreasing trend in isolates from Europe [23]. In the European Union/European Economic Area, the European Center for Disease Control data for 2023–2021 noted considerable differences in the percentages of CR strains, ranging from under 5% in two of 44 reporting countries to equal to or above 50% in 6 countries [24]. As recently as the first decade of this century, the absence of standardized international definitions of the degrees of multidrug resistance made it difficult to contrast the results of different studies, [25]. In 2012, a consensus document was published proposing international definitions of the terms multidrug resistance (MDR) (non-susceptible to ≥ 1 agent in ≥ 3 antimicrobial categories), extensive drug resistance (XDR) (non-susceptible to ≥ 1 agent in all but ≤ 2 categories) and pandrug resistance (PDR) (non-susceptible to all antimicrobial agents) [26]. However, in the case of *P. aeruginosa*, this proposal has certain limitations, since it was validated before the introduction of the novel β-lactam–β-lactamase inhibitor (BL-BLI) combinations, and the results vary depending on breakpoints used (The European Committee on Antimicrobial Susceptibility Testing—EUCAST or The Clinical & Laboratory Standards Institute- CLSI). Furthermore, the definition of XDR was formulated in terms of the number of antimicrobial categories with resistance but did not differentiate between first and second or third-line antimicrobial agents. In 2018, the IDSA (Infectious Diseases Society of America) proposed a new definition for pathogens with non-susceptibility to all first-line agents called Difficult-to-Treat Resistance (DTR), defined as resistance to all first-line (high efficacy and low toxicity) agents, which, in the case of gram-negative bacteria, meant resistance to fluoroquinolones and to all ß-lactams (including carbapenems) regardless of the susceptibility to the BL-BLI combinations and aminoglycosides [27]. Finally, a recent update by the European Study Group for Antimicrobial Resistance Surveillance of the European Society of Clinical Microbiology and Infectious Diseases suggests including 5 new categories, considering that neither the European Centre for Disease Prevention and Control nor the IDSA definitions take into consideration the newly approved β-lactams and BL-BLI combinations: ceftolozane/tazobactam, ceftazidime/avibactam, imipenem/relebactam, meropenem/vaborbactam and cefiderocol [28]. In the present review, we use as reference the term DTR *P. aeruginosa*. However, we maintain the original definitions of the studies named in the manuscript (CR, MDR and XDR) for a better interpretation of the outcomes.

The aim of the present research is to review the impact of multidrug resistance on bacterial virulence and fitness of *P. aeruginosa*. We discuss the microbiological implications, consequences for clinical outcomes and the economic burden of resistance in *P. aeruginosa*.

We try to make an integrative review of the resistance-virulence balance from all perspectives, from the basic and molecular research, through topics of molecular epidemiology and implications in large collections of clinical strains, until reaching patient cohorts and outcomes for them. To our knowldege there is no similar review in the literature, which allows to understand all the implications of resistance from the beginning (at the level of bacteria isolated in vitro) to the end (patient).

## Virulence factors in *Pseudomonasaeruginosa*

The virulence of a pathogen is its ability to infect the host and cause clinical symptoms, assisted by factors that promote bacterial adhesion, colonization, invasion of the host tissues, dissemination as well as evasion of the host immune response [31]. *P. aeruginosa* is endowed with a wide array of virulence factors that contribute to pathogenesis in acute and chronic infections [32], and regulated by complex, interconnected pathways and signaling systems that confer great plasticity to this pathogen [33]. Some of these virulence determinants are in the accessory genome acquired by horizontal gene transfer, which represents at least 10% of the genome, forming part of pathogenicity (PAPI) islands, such as PAPI-1 and PAPI-2 (responsible in part for the hypervirulence of strain PA14) or genomic (PAGI) islands [34–37].

Virulence determinants include cell-associated and extracellular factors. A list of the most important ones in *P. aeruginosa* is shown in Table 1.

**Table 1:** Main virulence determinants of *Pseudomonas aeruginosa*

Function	Categories	Description	References
*Outer membrane elements*			
Adhesion and colonization	Flagella	Bacterial adhesion, swarming and swimming motility, recognition by host cells or the immune system and biofilm formation	[232–234]
	Type IV pili	Attachment to host cells, twitching and swarming motility, DNA uptake and biofilm formation	[234–237]
*External surface structures (exopolysaccharides)*			
Adhesion and biofilm formation	Lipopolysaccharide	Adhesion to host cells and stimulation of immune response, interaction with TLR4 and CFTR, among other receptors. Resistance to complement and other antimicrobials	[238, 239]
	Pel and Psl	Bacterial adhesion, surface attachment and biofilm formation. Mainly produced by the strains obtained from the environment	[240–244]
	Alginate	Adhesion to host cells, immune evasion (inhibits phagocytosis), and biofilm formation. Generally secreted by strains isolated from cystic fibrosis patients	[240–244]
*Extracellular factors*			
Injection of effector proteins into host cells or extracellular secretion	Type 1 secretion system (T1SS)	Secretion of HasAp (siderophore), the alkaline proteases AprA and AprX and TesG (suppresses neutrophil response promoting chronic infections)	[247–249]
	Type 2 secretion system (T2SS)	Secretion of toxins (exotoxin A and pyocyanin) and exoproteins: lytic enzymes (lipases, proteases), elastases (LasA, LasB) which overcome host defense mechanisms	[247–249]
	Type 3 secretion system (T3SS)	Injection of the four effector proteins (ExoS, ExoT, ExoU, and ExoY) into the cytoplasm of host cells to disrupt intracellular signaling and induce apoptosis	[42, 251]
	Type 4 secretion system (T4SS)	Secretion of the proteins esterase (EstA), exoprotease (LepA) and hemagglutinin-like (CupB5)	[252, 253]
	Type 6 secretion system (T6SS)	Secretion of proteins associated with biofilm formation and environmental adaptation by delivering toxins to neighboring bacteria and translocation of effectors to host cells	[ 254, 255]
Siderophores	Pyoverdine and pyochelin	Iron uptake systems contributing to bacterial viability in iron-depleted conditions	[245, 246]
	Rhamnolipids	Degradation of alveolar surfactant, reduction of transepithelial electrical resistance and disruption of tight junctions in the respiratory epithelium	[256–258]
Bacterial cell- to-cell communication	Quorum-sensing (QS)	Regulation of gene expression that enables bacterial interaction and adaptation to environmental changes (including virulence factors). Biofilm formation	[147, 258–260]

*TLR4 *Toll-like receptor 4, *CFTR* cystic fibrosis transmembrane conductance regulator

Regarding cell-associated factors, on the bacterial surface there are appendages, such as flagella and type IV pili, which are the main cell adhesion and motility systems; and exopolysaccharides, also responsible for adhesion to host cells and biofilm formation (including the lipopolysaccharides: alginate and *Pel and Psl*).

Lipopolysaccharide is a major virulence factor present in many gram-negatives, comprised of lipid A (which anchors the structure in the outer membrane), core oligosaccharide, and O-antigen, which is the most distal element. This factor is a major structural component of the outer membrane, enabling resistance to the complement system, several antimicrobial peptides and toxic compounds, and exhibiting important pro-inflammatory effects. The O antigen has also been used to classify *P. aeruginosa* isolates [36–38] and certain variants are associated with a worse prognosis in respiratory tract (O1 and O11 serotypes) [40, 41] and bloodstream infections (O11 serotype) [29, 42].

Among the extracelular factors are included toxins, elastases, pigments, siderophores (pyoverdine and pyochelin) and other proteases. One of the most important *P. aeruginosa* virulence determinants is the type III secretion system (T3SS), which injects effector cytotoxins into host cells (ExoS, ExoT, ExoU and ExoY) [43, 44]. These exotoxins disrupt the epithelial barrier and so inhibit cell migration and proliferation, inducing apoptosis [45]. Of the four effector exotoxins identified, ExoU is the most potent as it leads to rapid necrosis of the epithelial barrier [44] and its expression has been associated with higher bacterial virulence and worse clinical outcomes in patients with pneumonia and bacteremia in both clinical and experimental studies [5, 38, 42, 46, 47]. The siderophores (pyoverdine and pyochelin) are produced by the bacterium as iron acquisition systems, vital for its growth and virulence [33, 48–51].

Exotoxin A is an ADP-ribosyl transferase that inhibits protein synthesis, inducing cell death, cytokine production, and reducing the host’s response to infection. LasA and LasB proteases exhibit elastolytic activity, apromoting degradation of blood vessel and pulmonary alveoli tissues. The alkaline protease and protease IV degrade complement proteins, cytokines (alkaline protease) and host clotting factors (protease IV). The rhamnolipids contribute to the pathogenesis of *P. aeruginosa* lung infections by degrading alveolar surfactant and participating in ciliostasis [33, 52–54].

Finally, quorum sensing, also secreted to the extracellular medium for bacterial cell-to-cell communication, involves the production, detection, and response to signaling molecules called autoinducers, that regulate gene expression, including a myriad of genes responsible for virulence-related behaviour. Thus, this system plays a key role in bacterial virulence, resistance, and biofilm formation [32].

## *Pseudomonasaeruginosa* epidemic high-risk clones

*Pseudomonas aeruginosa* shows significant clonal diversity, with more than 4,000 multi-locus sequence typing (MLST) profiles described (http://pubmlst.org/paeruginosa) [55]. However, this diversity primarily affects susceptible strains showing a polyclonal non-endemic pattern [56]. In contrast, most *P. aeruginosa* strains with a MDR/DTR phenotype, belong to a few endemic clones, known as high-risk clones [29, 30]. Multiple reports in recent decades have reported the worldwide spread of these clones [57–59], with clones ST235, ST111, and ST175 being the most prevalent globally [29]. These high-risk epidemic clones are characterized by their exceptional ability to acquire resistance determinants through horizontal gene transfer, especially horizontally-acquired-β-lactamases such as extended spectrum β-lactamases (ESBLs) and carbapenemases [28, 29, 60]. Table 2, adapted from del Barrio-Tofiño et al*.* [60], shows the main virulence characteristics, geographic distribution, and association with the main horizontally-acquired β-lactamases of the most prevalent high-risk clones.

**Table 2:** Characteristics of the main international *Pseudomonas aeruginosa* high-risk clones

Clone	Distribution	Typically associated horizontal β-lactamases	O-antigen serotype	Type III secretion system
ST235	Worldwide	Class A: GES, KPC Class B: FIM, IMP NDM, VIM Class D: OXA	O11	*exoU*+
ST111	Worldwide except Oceania	Class A: GES, KPC Class B: GIM, IMP, VIM	O12	*exoS*+
ST175	Europe, North America, and Asia	Class A: GES Class B: IMP, VIM	O4	*exoS*+
ST277	Europe, South America (predominantly Brazil), Asia and Oceania	Class A: KPC Class B: IMP, SPM	O2	*exoS*+
ST244	Worldwide	Class A: GES, KPC Class B: IMP, NDM VIM	O2	*exoS*+
ST233	Worldwide	Class A: KPC Class B: IMP, NDM, VIM	O6	*exoS*+
ST357	Europe, South America, Africa and Asia	Class B: IMP, NDM, VIM	O11	*exoU*+
ST308	Worldwide except North America	Class A: GES Class B: IMP, NDM, VIM	O11	*exoU*+
ST298	Europe, America, and Asia	Class B: IMP, VIM	O11	*exoU*+
ST654	Europe, America, and Asia	Class A: GES, KPC Class B: IMP, NDM, VIM	O11	*exoS*+

*References* [27, 29, 58, 60, 138, 262–264]

ST235 (O-antigen serotype O11) is considered the most significant high-risk clone with a wide distribution worldwide [29, 58] and is associated with over 60 different β-lactamase variants, including numerous Class A and B carbapenemases. ST111 (serotype O12) has also been documented on every continent except Oceania and is usually associated with the presence of metallo-β-lactamase (MBL) VIM-2. ST175 (serotype O4), is widely distributed in several European countries and Japan, and has a specific chromosomal mutation in AmpR (G154R) that causes hyperproduction of the intrinsic β-lactamase AmpC, OprD (Q142X), and MexZ (G195E), and 3 quinolone resistance-determining region (QRDR) mutations (GyrA T83I, D87N and ParC S87W) [58, 60–62]*.*

Other clones less extended are summarized in Table 2.

Different studies have evaluated the pathogenicity [60] and virulence of these high-risk clones [5, 64, 65]. Peña et al*.* determined that the T3SS genotype and high-risk clones are significantly linked, with specific high-risk clones showing well-defined T3SS genotypes [5]. Clone ST235 is known to have a greater capacity to acquire antibiotic resistance determinants and increased virulence [5, 66]. It is related with the *exoU* + virulence factor and the O11 serotype [28, 58, 67]. This clone has also been shown to be associated with increased mortality in bloodstream infections compared to other high-risk clones [47, 68]. On the other hand, other prevalent clones, such as ST111 and ST175, show the *exoU*-genotype and thus lower cytotoxicity, invasiveness, and virulence [62]. Recio et al*.* found that ST175 strains were associated with particularly low virulence, while the pathogenicity of ST235 strains was similar to that of non-MDR strains, despite exhibiting a wide range of acquired resistance mechanisms. Their findings also lend support to the T3SS genotype as one of the most important virulence determinants, since of the high-risk clones studied, ST235 was the only one with the *exoU*+ [69]. A Spanish multicenter study by Mulet et al., found that the three major high-risk clones (ST111, ST175, and ST235) were associated with low levels of virulence markers such as motility and pigment production, as well as reduced bacterial fitness, but also with increased spontaneous mutation frequency and biofilm growth, likely enabling a greater capacity for dissemination [70]. Other studies have demonstrated increased biofilm formation, high levels of pyocyanin and decreased motility in high-risk clones [71, 72].

As for the clones typically associated with cystic fibrosis, the most important and the first to be described was the Liverpool epidemic strain, or Clone C [73]. This clone has traditionally been considered non-virulent and differs by only 1 nucleotide from clone ST598 [74, 75]. Another clone widely distributed in patients with cystic fibrosis is ST274 (belonging to the clonal complex, CC274) [76]. This clone is associated with the presence of β-lactamases OXA-486 and PDC-24 genes, the O3 serotype and the *exoU*- genotype [77].

## Balance between antibiotic resistance and fitness/virulence in *Pseudomonasaeruginosa*: fundamental concepts and basic research

Fitness (also referred to as biological success) is the capacity of bacteria to survive and multiply within a specific niche and has traditionally been measured through growth rate and interbacterial competitive assays. When considering a pathogenic species such as *P. aeruginosa* causing acute infection, fitness is invariably linked to virulence, since the greater the capacity to infect and disseminate thanks to its virulence factors, the more likely it is to thrive inside the host, and vice versa [78, 79]. Given this close relationship, here we shall use fitness and virulence interchangeably, and the terms biological cost (or burden) and virulence attenuation as synonyms although we know that strictly speaking, they are not. Therefore, we will use them in a *sensu lato* conception to simplify the complex connection with resistance and make the reading easier. In other words, both if a bacterium grows slower in vitro or if it shows a very specific toxin hypo-expression for instance, the general outcome would be similar: an infection less harmful for the host. In any case, when in this section the consequences for biological success linked to each resistance mechanism are approached, we tried to deeply dissect them, clearly stating if attenuation is due to a general fitness impairment or linked to specific downregulations of pathogenesis-related features, which substracts importance of our indistinct use of concepts. However, it must be clarified that the analysis of the interplay between resistance and fitness/virulence we make here is referred to the acute infection context, because in the chronic infection (e.g. cystic fibrosis), this interplay is much more complex and particular. For instance, in this chronic scenario certain features such as slow growth and pathogenic attenuation are often positively selected, although these traits are deleterious if applied to acute infection [80]. Thus, the balance between antibiotic resistance and biological success in chronic respiratory infections is a very particular topic that would deserve an especially dedicated review and will not be considered in this manuscript.

Although it has been accepted for years that there is inevitably some fitness cost associated with acquired resistance in the context of acute infection, this assumption is not so clear-cut in reality [79, 81–83]. The view is based on the fact that an acquired mutation or horizontal determinant often has negative side effects for bacterial biology, such as alterations in antibiotic targets leading to decreased protein efficiency, increased energetic costs associated with the (hyper)production of enzymes that inactivate the antibiotic, unwanted extrusion of certain useful compounds in addition to the drug, reduced nutrient uptake because of porin loss, etc. This assumption justifies the research interest in looking closely at resistance and fitness with the ultimate goal of discovering therapeutic targets to reduce the pathogenic power of bacteria and/or hinder the emergence of resistance by taking advantage of the potentially high associated biological costs [30, 84]. However, as will be seen below, there are multiple examples of cases where there appears to be no biological burden, either because the acquired mechanisms themselves appear to be cost-free, or compensatory mutations are selected, or even because other markers of increased virulence are co-carried with the acquired resistance gene on mobile genetic elements [81, 85, 86]. There are several studies with clinical strains or mutants evolved in vitro, in which the resistance phenotype is treated as the end result of a combination of mechanisms, but it is very difficult to establish direct cause-effect relationships between specific resistance pathways and biological costs in these studies. In this section on the other hand, we review the resistance/fitness trade-off from a molecular perspective, collecting data in which different resistance mechanisms and their respective biological burdens are addressed separately. Thus, here we take a close look at the main knowledge in the field of basic research on the connection between resistance and fitness/virulence in *P. aeruginosa*, dividing the topic into horizontally-acquired versus mutation-driven resistance for ease of understanding. Subsequently, the clinical perspective of the interplay between resistance and virulence in *P. aeruginosa* will also be addressed to provide an integrated overview of the topic.

### Horizontally-acquired resistance

It has to be considered that the biological cost associated to horizontal resistance genes may be often due to the the carrier elements (usually plasmids) themselves rather than to the specific resistance determinant. However, since multiple factors could determine the cost of each particular plasmid [87–89] (size, copy number, codified genes, or even compensatory features such as pathogenicity islands, etc.), to simplify the approach in this section we dissect the knowledge about the biological burden, apparently specific, associated to the main horizontally-acquired determinants.

The paradigmatic example of horizontally-acquired resistance is the incorporation of β-lactamases—typically encoded by genes in class I integrons—carried on mobile elements such as plasmids, although few studies have specifically analyzed their associated biological costs. The results in this regard are variable, ranging from β-lactamases for which no associated attenuation was observed to others manifesting significant measurable impairment of fitness/virulence linked to certain enzymes. Among the former, some examples can be cited, such as the class B carbapenemases IMP-1 and VIM-1, the class A penicillinase PSE-1, the class A extended-spectrum β-lactamase [ESBL] GES-1, the class C cephamycinases FOX-4 and FOX-8, and the narrow spectrum class D OXA-2 enzyme [90–92] Conversely, other β-lactamases have been shown to entail important burdens, such as the case of class A TEM-1 and class D OXA-3 enzyme production, which triggered impaired twitching motility, adhesion, and biofilm formation by *P. aeruginosa* without affecting its growth [93]. More recently, production of the OXA-2-derived ESBLs, OXA-226, OXA-161 and especially OXA-539, was shown to be associated with significantly attenuated virulence in an invertebrate model [92]. Although the underlying basis for these high burdens has not been fully determined, some possible explanations have been advanced. For instance, given the likely common origin with ancestral enzymes showing peptidoglycan-lytic activity, it was proposed that some β–lactamase variants could still display certain capacity to metabolize the own cell wall nowadays [87, 94]. Thus, in addition to hydrolyzing the β–lactam drug, in certain circumstances these β-lactamases could be weakening the cell wall by partially degrading it, which would impair cell viability, obviously entailing a great biological burden. Moreover, this residual activity could affect the peptidoglycan-derived molecules, altering the pool of soluble fragments and triggering a downregulation of pathogenesis through appropriate regulators, similar to what happens with AmpR and the muropeptide-linked modulation of AmpC β-lactamase expression [95]. The fact that a functional active site of TEM-1 was required to cause the abovementioned biological burden associated to its production [93], would support the idea of a residual peptidoglycan-degrading activity being the key for this outcome, although other explanations have not been ruled out.

From the abovementioned data, it can be concluded that most of the relevant horizontally-acquired β–lactamases have not yet been analyzed from the perspective of their impact on fitness/virulence. However, considering the worldwide epidemic dissemination of certain enzymes (including OXA-10, PER-1, NDM-1, KPC-2, VIM-2, certain GES-type ESBLs/carbapenemases, among many others), which are often associated with successful high-risk clones, it may be assumed that their costs must be low overall if these associated costs were high, these enzymes would not be so globally widespread [28, 60]. By the same token, a very high cost linked to specific amino acid variants (the aforementioned OXA-539 for instance) [92] would explain their lack of epidemiological success despite the increased capacity for β–lactam hydrolysis. In any case, the possibility of some connections between certain high-risk clones and quite specific β–lactamases being caused by particular traits of the strain but not by the absence of biological cost associated to each β–lactamase per se has not been ruled out. Thus, the opposite phenomenon cannot be discarded either, i.e. that some β-lactamase variants such as the abovementioned OXA-2 derived ESBLs could globally disseminate in future if they get to be acquired by high-risk clones in which the fitness costs of these enzymes may be minor. Although this interesting topic has been approached in *Escherichia coli* [96], and was shown to explain why some β-lactamases are seemingly limited to certain species and absent in others (e.g. VIM-2 and SPM-1 in *Acinetobacter baumannii* [97]) remains to be investigated in *P. aeruginosa*, posing an interesting field worth to be opened.

Athough besides β-lactamases the acquisition of aminoglycoside-modifying enzymes in class I integrons is a typical hallmark of *P. aeruginosa* clinical strains [98], the topic of their potential biological costs has been never investigated in this species. Therefore, although a presumable low burden can be deduced (if it was high, these enzymes would not be that disseminated) whether these determinants entail different costs depending on the family (N-acetyltransferases, O-nucleotidyltransferases and O-phosphotransferases) and/or the host strain are questions that remain to be approached in future. Conversely, recent work has studied the biological cost associated with the horizontally-acquired colistin resistance determinant, Mcr-1 (in which the addition of phosphoethalonamine to lipopolysaccharide reducing colistin affinity for its target), revealing a lack of impact on fitness/virulence in *P. aeruginosa* [99]. This represents a red flag for the potential dissemination of this determinant in *P. aeruginosa* (at the moment, this mechanism is much more prevalent in *Enterobacteriaceae*), threatening the future antipseudomonal effectiveness of this drug of last resort.

### Mutation-driven resistance

With respect to mutation-driven resistance, probably the most important mechanism in *P. aeruginosa* is hyperproduction of chromosomal AmpC cephalosporinases, which affects 3rd- and 4th-generation cephalosporins, as well as antipseudomonal penicillins and monobactams. Different mutations in genes related to peptidoglycan metabolism cause this hyperproduction [48], with no demonstrable biological burden associated with the two most common pathways, namely, disruption of the *ampD* and *dacB* genes [100]. On the other hand, when AmpC hyperproduction was combined with presence of a PSE-1 enzyme in a murine infection model, dramatic attenuation was reported [91], suggesting that AmpC hyperproduction in itself is a very potent, cost-free resistance mechanism and may have deleterious effects only when combined with certain other circumstances [91, 100]. In this context, it was recently shown that the burden of AmpC hyperproduction occurring only in very particular scenarios (peptidoglycan recycling blockade) is dependent on enzymatic activity and reinforces the idea that the abovementioned residual *peptidoglycanase* activity in certain β-lactamases may be a key contributor to impaired fitness/virulence [92, 100–102]. In fact, this need for an intact active site had been already shown for TEM-1 [93], supporting the idea of a partial degradation of the own murein sacculus due to this residual activity, as the cause of cell viability impairment and the necessarily derived fitness cost. Obviously, blocking an essential pipeline for the production of new material to be incorporated into nascent peptidoglycan, as are the recycling routes, would further weaken the cell wall revealing or exaggerating the biological costs [100, 101]. These outcomes could be also caused by the residual *peptidoglycanase* activity acting on a presumptive pathogenesis regulatory muropeptide pool [95] and not only on the peptidoglycan as rigid structure, as abovementioned for certain horizontally acquired β-lactamases [92, 93].

As a result of the selective pressure exerted by the new drug combinations ceftolozane/tazobactam and ceftazidime/avibactam, there are increasing reports of certain amino acid variants appearing in chromosomal AmpC causing resistance to these antimicrobials when combined with hyperproduction [103]. Of these, only the T96I, G183D and ΔG229-E247 variants have been analyzed from a fitness/virulence perspective and have been shown not to increase costs relative to wildtype AmpC values [100]. In contrast, hyperproduction driven by an amino acid change (G154R) in the master AmpC regulator, AmpR, has been shown to contribute to reduced virulence in the *Caenorhabditis elegans* model, which is consistent with the fact that AmpR apparently governs the expression of certain virulence-related genes [104, 105]. However, taking into account that this AmpR variant is very closely linked to the high-risk clone ST175 (see "Pseudomonas aeruginosa epidemic high-risk clones" section), it is likely that the increased resistance to β–lactams that it affords more than compensates for the associated cost.

The mutation driven loss of the OprD porin, which confers increased resistance to carbapenems, has been associated with remarkably hypervirulent behavior, including increased cytotoxicity towards macrophages and lethality in mice, although the underlying basis for this phenomenon has not yet been resolved [106, 107]. Other studies of the same mechanism in murine models however have shown a slight reduction in fitness [91, 108], suggesting that the contradictions in these studies may be due to differences in the *P. aeruginosa* strains used and the fitness/virulence-related parameters analyzed.

Hyperexpression of efflux pumps is one of the most important resistance mechanisms in *P. aeruginosa* and affects practically all antipseudomonal drugs. The major efflux pumps in this context are MexAB-OprM, MexCD-OprJ, MexEF-OprN and MexXY-OprM, forming a complex modulatory system made up of different regulators with mutation profiles driving hyperproduction in each of them [109]. From the point of view of fitness/virulence, MexCD-OprJ hyperproduction, mediated by mutations in its *nfxB* repressor, seems to be clearly associated with attenuated virulence [110–113]. In the case of MexEF-OprN hyperexpression, deletion of this pump resulted in a corresponding increase in virulent behavior, with improved collagenase activity, rhamnolipid production and swarming motility, although the underlying basis of this hypervirulence is not fully understood [111, 114–121].

The information relating to MexAB-OprM is conflicting, with some data showing a significant biological burden [122, 123] contrasting with other results in which pump hyperexpression was associated with enhanced virulence [119, 124]. Of a similar contradictory nature, both MexXY-OprM hyperexpression and its deletion led to significant impaired virulence, including cell invasiveness and pyoverdine production, which once again demonstrates the complexity of the interplay between resistance and virulence [125, 126]. In general, attenuated virulence in the aforementioned pumps has been related to decreased release of extracellular factors such as proteases, elastase, pyocyanin and rhamnolipids (linked in turn to swarming motility) as well as impaired performance of the T3SS. A common explanation for the attenuated virulence often reported to be associated with hyperexpression of efflux pumps is that, in addition to extrusion of the antibiotics, the pumps are also expelling the quorum sensing signals (and thus decreasing their intracellular concentrations) needed for efficient production of different virulence factors [110–116, 118–120, 122, 123, 125].

DNA gyrase consists of two subunits encoded by the *gyrA* and *gyrB* genes, whereas the topoisomerase IV subunits are encoded by *parC* and *parE*. These proteins, which are involved in the DNA relaxation/coiling processes necessary for replication, are the targets of quinolones. Hence, certain amino acid changes in their sequences are associated with lower binding affinity of the drug for their targets and related resistance, which explains why they are known as Quinolone Resistance-Determining Regions (QRDR) [127]. The burden associated with certain QRDR mutations has been studied, and it has been shown that, in general, low levels of quinolone resistance are mostly cost-free, whereas the highest levels require sequential mutations in the mentioned genes, which obviously leads to increased biological costs. These have been linked to alterations in DNA supercoiling levels, consistent with impaired DNA gyrase and/or topoisomerase IV action [128]. However, it has been shown that the burden associated with QRDR mutations can be readily alleviated by compensatory mutations at different sites [83, 128]. Interestingly, it has been suggested that the T3SS toxin ExoU may better regulate DNA supercoiling, which relieves the burden associated with QRDR mutations [129], and would explain the reported correlation between the presence of this toxin and the high prevalence of QRDR mutations in *exoU*+ clinical strains [130].

In *P. aeruginosa*, the main mechanism of colistin (polymyxin E) resistance is the addition of 4-amino-4-deoxy-l-arabinose to lipopolysaccharide, which is associated with mutations in different two-component systems leading to overexpression of the *arnBCADTEF* operon, which encodes aminoarabinosylation enzymes [131]. It was recently shown that aminoarabinosylation in itself (achieved by cloning and constitutive hyperexpression of the enzymes involved) has a negligible impact on *P. aeruginosa* fitness and virulence [132]. However, it has also been shown that after 21 days of exposure to colistin in a morbidostat device, resistant mutants displayed other side effects such as improved serum resistance and biofilm formation, but decreased *insect larvae* killing capacity, demonstrating the complex implications of colistin resistance [133]. Closely related to colistin and in this same latter sense, it was shown that polymyxin B resistance development (whose mechanisms were not determined) was associated with a significant attenuation of *P. aeruginosa* virulence in the invertebrate model used, but this outcome was only seen in a DNA repair-deficient hypermutator strain (*mutS* KO mutant), not in the wildtype background [134].

A final example that contradicts the oversimplistic view that resistance usually carries biological costs is the mutational disruption of *glpT* (that encodes the transporter that enables fosfomycin uptake) which had no measurable effect on impaired fitness or even an increased pathogenic response, in parallel to fosfomycin resistance [106, 135, 136].

## Balance between antibiotic resistance and fitness/virulence in *Pseudomonasaeruginosa*: collections of clinical strains and animal models

In the previous section we reviewed the interplay between resistance and biological burden from a molecular perspective, focusing on specific resistance mechanisms. In this section, we review other studies that have analyzed the interplay between resistance profiles and virulence in clinical strains from the more general point of view of resistance as the final outcome (not considering specific mechanisms). In this section, we consider both in vivo models and in vitro studies performed with clinical strains of *P. aeruginosa.*

### Clinical strains

Focusing on the clinical strains, many authors have attempted to clarify the association between different virulence factors and MDR/XDR profiles. Studies are summarized in Table 3.

**Table 3:** Studies reviewing the interaction between resistance profiles and virulence in clinical strain collections and through animal models

Author, year, reference	Infection type	Study design	Study population	Study objective	Main findings
*Clinical strains*					
Gajdács et al., 2022, [146]	Multiple sample types	Multicenter retrospective cohort study	Hungary, Italy (302 clinical isolates, 133 MDR)	To establish the relationship between biofilm-forming capacity, the expression of some important virulence factors, and the MDR phenotype	MDR and non-MDR isolates did not show significant differences in expression of virulence factors, with the exception of pyocyanin production (MDR: 0.371 ± 0.193 vs. non-MDR: 0.319 ± 0.191; *p* = 0.018) No relevant correlations were seen between biofilm formation, pigment production, or motility
Ratajczak et al., 2021, [149]	Multiple sample types	Single center retrospective cohort study	Poland (73 clinical isolates)	To assess the antibiotic resistance, biofilm formation, and the presence of genes encoding virulence factors	The biofilm-forming ability was significantly higher among the MDR strains (*p* = 0.021)
Yamani et al., 2021, [151]	Multiple sample types	Single center retrospective cohort study	Saudi Arabia (66 clinical isolates, 40 MDR)	To assess the biofilm profile and to determine whether there is a correlation between antimicrobial resistance patterns and biofilm formation capacity	The biofilm production capacity was significantly higher among the non-MDR isolates (*p* = 0.002)
Eladawy et al., 2021, [150]	Multiple sample types	Single center retrospective cohort study	Egypt (103 clinical isolates)	To investigate the correlation between biofilm formation and antimicrobial resistance and the presence of virulence genes	There was no significant correlation between biofilm formation and individual antibiotic resistance in all isolates
Recio et al., 2020, [189]	BSI	Single center retrospective cohort study	Spain (243 clinical isolates)	To explore the prognostic factors affecting mortality emphasizing on antimicrobial resistance and virulence	O4 and O11serotypes were more frequently associated with the MDR phenotype, especially the XDR phenotype The O11 serotype was more frequently associated with the *exoU*+ genotype, and the O4 and O6 serotypes with the *exoU*-genotype
Deptula et al., 2020, [141]	Multiple sample types	Single center retrospective cohort study	Poland (150 clinical isolates: 75 MDR)	To compare phenotypic differences in the expression of virulence factors	MDR strains showed lower adhesion to polystyrene than non-MDR strains (0.111 vs. 0.130; *p* = 0.005), lower growth rate in liquid medium (0.924 OD vs. 1.234 OD; *p* < 0.001), and lower activity of elastase (*p* < 0.01), LasA protease (*p* < 0.001), lipolytic (*p* < 0.001) and phospholipase C activity (*p* < 0.001) MDR strains produced significantly lower amounts of extracellular material binding Congo Red (*p* = 0.02) and less pyocyanin (*p* < 0.001) No significant differences were observed between MDR and non-MDR strains in proteolytic activity
Del Barrio-Tofiño et al., 2019, [138]	Multiple sample types	Multicenter retrospective cohort study	Spain (1445 clinical isolates)	To evaluate the correlation of O-antigen serotypes with resistance profiles and high-risk clones	The most frequent serotype among XDR isolates was O4 (34.1%), followed by O11 (15.9%) Within serotypes, XDR phenotypes were more frequent for O12 (60.0%) and O4 (57.3%) ST175 was associated to O4, CC235 and ST308 were associated with O11, whereas CC111 was linked to O12
Horna et al., 2019, [137]	Multiple sample types	Multicenter retrospective cohort study	Peru (189 clinical isolates)	To determine the presence of the* exoU*+/*exoS*+ genotype and its association with phenotypic characteristics, resistance genes related to MDR and efflux pump regulators	The* exoU*+/*exoS*+ genotype was associated with MDR and XDR phenotypes No association between the* exoU*+ genotype and motility (swarming and swimming) or biofilm formation The presence of* exoU* + was associated with higher level of quinolone resistance
Rodulfo et al., 2019, [143]	Multiple sample types	Single center retrospective cohort study	Venezuela (176 clinical isolates)	To evaluate how the virulence factors, resistance genes and integrons are affecting the epidemiological outcome of PA	MDR/XDR strains were positively associated with hemolysins and* exoU*+, but negatively associated with bacterial twitching Production of DNases, gelatinase, biofilm and pigment was not statistically associated with the level of resistance
El-Mahdy et al., 2019, [144]	Multiple sample types	Single center retrospective cohort study	Egypt (80 clinical isolates, 58 MDR or XDR)	To investigate the rate of carbapenemases genes, antibiotic resistance, and virulence factors in CR PA associated with hospital-acquired infections	Biofilm formation was significantly associated with CR isolates (*p* < 0.05) Pyocyanin production was significantly correlated to carbapenem susceptible isolates (*p* < 0.05)
Kaiser et al., 2017, [147]	Multiple sample types	Single center retrospective cohort study	Germany (248 clinical isolates: 211 XDR and 38 non-MDR)	To determine which determinants facilitate the spread persistence of MDR and XDR PA in hospital settings	XDR isolates showed significantly elevated biofilm formation (*p* < 0.05) and higher competitive fitness compared to non-XDR XDR group showed significantly elevated serum susceptibility
Fuse et al., 2013, [142]	Multiple sample types	Multicenter retrospective cohort study	Japan (50 clinical isolates: 20 MDR)	To investigate the pyocyanin producing ability of MBL-producing MDR PA	MDR strains produced a significantly smaller amount of pyocyanin (*p* < 0.05)
Finlayson et al., 2011, [145]	Multiple sample types	Single center retrospective cohort study	Jamaica (57 clinical isolates)	To determine differences in the virulence factors of pigmented and non-pigmented PA isolates	Pigmented isolates produced more frequently and significant more (*p* < 0.05) DNase, elastase, lipase protease, and siderophore No significant differences were found in antibiotic resistance between pigmented and non-pigmented isolates
Karatuna et al., 2010, [148]	Respiratory isolates	Single center retrospective cohort study	Turkey (100 clinical isolates)	To assess the activity of QS-dependent virulence factors in respiratory PA isolates and their relationship with antimicrobial susceptibility	Statistically significant correlations were observed between: The lack of elastase production and resistance to piperacillin and ceftazidime The failure in alkaline protease production and resistance to tobramycin, piperacillin, piperacillin-tazobactam, cefepime, imipenem and ciprofloxacin The failure in pyocyanin production and resistance to amikacin, tobramycin, ceftazidime, ciprofloxacin and ofloxacin
Jamasbi et al., 2008, [139]	Multiple sample types	Single center retrospective cohort study	USA (167 clinical isolates)	To determine the frequency and distribution of the serotypes	MDR phenotype was ssociated with serotypes O2, O4, O11 and O15
*Animal models*					
Pincus et al., 2020, [159]		Experimental bloodstream infection murine model	USA (115 clinical isolates)	To assess whether it was possible to predict the virulence of isolates based on their genomic content	Machine learning models trained using accessory genomic elements were predictive of virulence, with a mean nested cross validation accuracy of 75% using the random forest algorithm Machine learning models trained using core genome single-nucleotide variants and whole-genome k-mers (overlapping sequences of length k) also predicted virulence
Recio et al., 2020, [69]		Single center retrospective cohort study	Spain (38 clinical isolates: 21 MDR)	To investigate the interplay between antimicrobial resistance and virulence, emphasizing on MDR ST175 and ST235 high-risk clones in a *Caenorhabditis elegans* model	MDR strains showed lower cytotoxicity (35.4 ± 21.30% vs. 45.0 ± 18.78%; *p* = 0.044) and virulence (66.7%vs. 100%;* p* = 0.011) than non-MDR strains ST235 showed the highest cytotoxicity and ST175 the lowest (51.8 ± 10.59% vs. 11.0 ± 1.25%; *p* < 0.0001) ST235 clone showed a trend towards higher virulence (100% vs. 73.1%; *p* = 0.075), whereas ST175 strains showed the lowest virulence (0% vs. 93.9%; *p* < 0.0001) Virulent strains were significantly more cytotoxic than nonvirulent strains (11.5 ± 2.31% vs. 46.1 ± 17.28%; *p* < 0.0001)
Sánchez-Diener et al., 2020, [167]		Multicenter prospective cohort study	Spain (593 BSI isolates)	To assess the association between the lethality of PA in a *Caenorhabditis elegans* model and outcomes	No association between virulence phenotype and 30-day mortality was found (28.7% vs. 28.5%; *p* = 1) An inverse association between *C. elegans* virulence and MDR and XDR profiles was documented (aOR 0.655 95% CI 0.571–0.751) and (aOR 0.523 95% CI 0.436–0.627); *p* < 0.001, respectively) The* exoU* genotype was significantly more frequent among isolates showing high virulence (19.9% vs. 25.1%, *p* < 0.001)
Sánchez-Diener et al., 2017, [104]		Multicenter retrospective cohort study	Spain (140 isolates: 80 BSI, 20 XDR, 20 MDR, 20 ModR, 20 epidemic XDR isolates)	To decipher the interplay among resistance profiles, high-risk clones and virulence	A clear inverse correlation between antimicrobial resistance and virulence in the *C. elegans* model was documented The lowest virulence was linked to XDR profiles, which were typically linked to defined high-risk clones ST235 was found to be linked with higher virulence, while ST175 was associated with a particularly low virulence
Gómez-Zorrilla et al., 2017, [157]		Experimental murine peritonitis-sepsis model	Spain (9 strains: 7 clinical strains, 2 reference strains)	To investigate how the inflammatory response occurs in the most relevant high-risk clones and to compare the process with that recorded in clinical susceptible isolates	TNFα and IL-10 levels were significantly higher at all time points in mice inoculated with non-MDR strains IL-6 levels were significantly higher in the non-MDR group at 8 h and 12 h Bacterial counts in peritoneal fluid were higher in the non-MDR group compared at 8 and 12 h
Gómez-Zorrilla et al., 2016, [64]		Experimental murine peritonitis model	Spain (9 strains: 7 clinical strains, 2 reference strains)	To evaluate the relationship between pathogenicity and the resistance profile of different PA strains, including the most common epidemic high-risk clones	The in vitro bacterial duplication time was shorter in clinical non-MDR strains (0.42 ± 0.08 h vs. 0.55 ± 0.14 h; *p* = 0.023) In the animal model, the probability of mortality at 48 h was 70% for clinical non-MDR strains versus 7.5% for MDR (*p* < 0.001) Bacterial concentrations in peritoneal fluid were higher in mice inoculated with non-MDR (*p* < 0.001)
Giamarellos-Bourboulis et al., 2011, [156]		Experimental empyema model in rabbits	Greece (2 clinical isolates: 1MDR)	To define the impact of MDR in experimental lung infection	The susceptible isolate produced higher mortality than the MDR isolate (*p* = 0.041)
Giamarellos-Bourboulis et al., 2004, [154]		Experimentalsepsis model in rabbits	Greece (3 BC isolates: 2 MDR)	To evaluate whether susceptible and MDR phenotype differ in the mechanism of induction of sepsis	Mortality was higher in in infections caused by susceptible strains than MDR Bacterial load was higher in animals inoculated with susceptible strains than MDR strains
Giamarellos-Bourboulis et al., 2004, [155]		Experimental murine peritonitis- sepsis model	Greece (20 clinical isolates, 12 MDR)	To investigate the stimulatory effect of PA on innate immunity and to correlate it to its level of resistance to antimicrobials	Susceptible isolates induced a statistically significant increased release of IL-1b, IL-6 and MDA by human monocytes compared to MDR isolates Progression to death evolved rapidly in rats challenged by susceptible isolates compared to those challenged by MDR isolates

BC, blood cultures; BSI, bloodstream infections, CC, clonal complex; CI, confidence interval, CR, carbapenem-resistant, aHR, adjusted hazard ratio, IL, interleukin, MBL, metallo-b-lactamase, ModR, moderately resistant (nonsusceptible to at least one agent in 1 or 2 classes); MDR, multidrug-resistant; aOR, adjusted odds ratio; PA, *Pseudomonas aeruginosa*; QS, quorum sensing; TTSS, type III secretion system; ST, sequence type; XDR, extensively drug-resistant

As mentioned above, several studies with clinical strains suggest that the T3SS genotype, [5, 69, 137] and the serotypes [42, 138, 139], are key virulence factors in *P. aeruginosa.* Particularly, the *exoU* genotype is the major virulence determinant of *P. aeruginosa* [5, 38, 42, 46, 47, 69, 140].

Different studies have explored the relationship between other virulence determinants and antimicrobial resistance phenotypes reaching different conclusions. Regarding the ability to produce pigments such as pyocyanin some studies found a negative correlation between MDR profile and pyocyanin production [141, 142] while others did not find association between the resistance phenotype and the pyocyanin production [70, 143–145]. Other virulence determinants such as motility or alginate production were also analysed, but contradictory results were observed regarding the association of these virulence determinants and the resistance pattern [141, 144–146] as it is shown in Table 3.

With specific reference to the activity of virulence factors conventionally described as highly dependent on quorum sensing (elastase, alkaline protease, pyocyanin and biofilm production), in a single center restrospective study was analyzed the relationship between these and antimicrobial susceptibility in respiratory isolates and was found that those that tested negative for virulence factor production were predominantly more resistant to antimicrobials [148].

Finally, numerous studies have analyzed the relationship between biofilm production and resistance with some studies that found a correlation between MDR/XDR resistance profile and a higher ability to biofilm formation [144, 147, 149] and others showing no correlation between the resistance profile and the biofilm formation [143, 146, 150] or even a negative correlation [151].

These conflicting results in the resistance profiles of clinical strains one more time highlight the significant complexity in the relationship between resistance and virulence.

### Animal models

In vitro investigations of human pathogens do not always replicate natural conditions, and the expression of virulence factors is not the same when cultured in vitro as in animals or humans. As a result, animal models have become a valuable tool for advancing our knowledge of *P. aeruginosa* pathogenesis and host–pathogen interactions as well as for the development and evaluation of new therapies [152, 153]. Although some studies involving animal models have been already mentioned in previous paragraph, in this section we bring together the most important studies dealing with virulence-resistance interplay in *P. aeruginosa,* in which animal models play a key role.

With respect to vertebrate models, Giamarellos-Bourboulis et al*.* have used experimental models observing higher mortality, stimulation of innate immunity and bacterial load in infections caused by susceptible strains than by MDR strains [154–156].

The authors suggested that their findings may support the idea that some of the differences in virulence between MDR and susceptible *P. aeruginosa* can be attributed to interactions with the innate immune system. Similar results were found by Gómez-Zorrilla et al. [64, 157].

In general, susceptible *P. aeruginosa* strains induced a higher inflammatory response than MDR strains, but even strains with a similar resistance phenotype induced different responses. Although their data suggested that there was a fitness cost associated with multidrug resistance, the magnitude of the inflammatory response was the result not only of the resistance phenotype but also of the virulence phenotype. The expression of virulence factors not only affects the virulence of *P. aeruginosa* but also alters the magnitude of the host–pathogen interaction, accounting for the differences in the elicited inflammatory response [157].

Recently, a study has evaluated the usefulness of a machine learning approach to predict *P. aeruginosa* virulence in a mouse model of bloodstream infection based on genomic content, using a training set of *P. aeruginosa* bacteremia isolates. Authors showed that the core genome, alone or in combination with the accessory genome, is predictive of virulence. Thus, the machine learning analyses performed in this study could serve as a framework for further investigation of the relationship between bacterial genomes and their phenotype [159].

As an alternative to the vertebrate models, studies are increasingly being conducted in invertebrate models such as the fruit fly *Drosophila melanogaster*, the nematode *Caenorhabditis elegans*, and the wax moth *Galleria mellonella* [160, 161]. *C. elegans* studies are a promising model for studying developmental biology and host–pathogen interactions [162–165]. In this regard, Recio et al*.* found that the virulence of the *P. aeruginosa* strains they studied correlated significantly with the non-MDR and MDR phenotype, *exoU*+ and *exoS*+ genotypes, O4 and O11 serotypes and the ST175 high-risk clone [69]. Sánchez-Diener et al. documented a clear negative correlation between antimicrobial resistance and virulence by testing a large collection of well-characterized *P. aeruginosa* isolates from different sources (bloodstream infections, nosocomial outbreaks, cystic fibrosis and environmental samples) in a *C. elegans* infection model. The lowest virulence was linked to XDR profiles, but virulence varied considerably depending on the high-risk clone involved, being high for ST111 and ST235, but very low for ST175. The highest virulence of ST235 could well be attributed to its *exoU* + T3SS genotype, linked to higher virulence in the *C. elegans model,* as well as in previous clinical studies [104]. Therefore, they suggested that the global transcriptional regulator AmpR could be involved in modulating bacterial pathogenicity as shown in previous works [105, 166]. The same authors investigated the impact of virulence phenotype on mortality in 593 *P. aeruginosa* bloodstream isolates recovered from a prospective Spanish multicenter study in order to assess the predictive value of *C. elegans* lethality as a prognostic marker. Their results indicated that the virulence phenotype of *P. aeruginosa* (specifically in the *C. elegans* model) correlated with virulence genotype (T3SS) and resistance profile but was a poor prognostic marker of mortality in bloodstream infections [167]. On the other hand, previous studies by Miyata and collaborators determined that the greater wax moth caterpillar *G. mellonella* is also a suitable invertebrate model host for studying the role of T3SS in *P. aeruginosa* pathogenesis [161]. Moreover, a very good correlation in the virulence outputs showed by 32 different *P. aeruginosa* strains when comparing *G. mellonella* with murine models was also found in another study, demonstrating the validity of this invertebrate model [168].

## Clinical impact of multidrug resistance in *Pseudomonasaeruginosa* infections

The clinical impact of multidrug resistance in *P. aeruginos*a is not only determined by bacterium-related factors, as reviewed in the sections above, but also by factors related to antimicrobial therapy, as well as host-dependent factors such as immune response. Against this background, it is difficult to determine the exact direct relationship between multidrug resistance and clinical outcomes. While it is generally assumed that infections caused by MDR microorganisms are associated with worse outcomes [169–172], these worse outcomes could be associated with treatment and/or host factors, rather than factors directly associated with bacteria.

With respect to the treatment, one of the main consequences of MDR/DTR infections is the challenge of selecting an appropriate empirical antibiotic therapy. Infections caused by MDR/DTR *P. aeruginosa* are associated with an increased risk of the patient receiving inappropriate empirical therapy and delayed appropriate antimicrobial therapy [173, 174]. It is well demonstrated that delayed appropriate empirical antimicrobial therapy and decreased antibiotic effectiveness are associated with increased mortality [1, 12, 175–177]. In addition, antimicrobial therapies used for MDR/DTR infections may be less effective than those used to treat susceptible infections, often requiring second-line antimicrobial agents or suboptimal treatments [178].

With respect to the host, MDR/ DTR-*P. aeruginosa* infections usually occur in severely ill patients with multiple underlying diseases, frequently associated with longer hospitalization or previous ICU admissions. These factors may explain, at least in part, the worse outcome of MDR/DTR-infections [179–182]. In one of the largest studies evaluating the impact of carbapenem resistance on 30-day mortality, Peña et al. demonstrated that carbapenem resistance was associated with significantly increased 30-day mortality rates but that the relationship depended on the degree of baseline severity [183]. Thus, patients with infections due to multidrug-resistant microorganisms tend to have more comorbidities, which may lead to higher mortality [12].

Although a number of studies over the years have compared the different outcomes for infections caused by susceptible and multidrug-resistant strains, the question of whether infections caused by multidrug-resistant microorganisms lead to worse outcomes than those caused by susceptible ones is still being debated. Table 4 lists and summarizes the main previous studies conducted to evaluate differences in clinical outcome between susceptible and drug-resistant *P. aeruginosa* infections according to source of infection [bacteremia, pneumonia, and urinary tract infection (UTI)], there are conflicting results with some studies suggesting that MDR infections are associated with increased mortality and others showing the opposite.

**Table 4:** Clinical studies providing outcome information for infections due to MDR/DTR *Pseudomonas aeruginosa* (first studies supporting association of resistance with mortality and second studies against the association)

Author, year, reference	Infection type	Study design	Study population (country, no., condition)	Study objective	Main findings
Herrera et al., 2023, [184]	BSI	Single center retrospective cohort study	Spain (2057 episodes: 475 CR PA)	To describe the clinical characteristics and outcomes of PA BSI and analyze whether transplantation could be a risk factor for MDR infections and/or mortality	CR phenotype was associated with higher 30-day mortality (aOR 1.53, 95% CI 1.01–2.29; *p* = 0.036) in the overall cohort Hematologic malignancy (aOR 2.71, 95% CI 1.33–5.51; *p* = 0.0069) and prior carbapenem therapy (aOR 2.37 95% CI 1.46–3.86; *p* < 0.001) were associated with higher risk of having a CR PA BSI Solid organ transplantation was not associated with higher risk of CR PA BSI
Yuan et al., 2023, [185]	BSI	Multicenter retrospective cohort study	China (274 patients: 137 CR PA, 93 MDR, 46 DTR)	To analyze the risk factors and outcomes of CR PA BSI	The 30-day survival probability of patients with CR, MDR and DTR PA BSI was significantly worse than for those with susceptible phenotype (aHR 1.73, 95% CI 1.11–2.68; *p* = 0.014; aHR 2.30, 95% CI 1.45–3.646; *p* < 0.001 and aHR 2.13, 95% CI 1.14–4.00; *p* = 0.002; respectively)
Zhen et al., 2023, [186]	BSI	Single center retrospective cohort study	China (100 CR PA, 45 MDR, 7 DTR)	To evaluate mortality at day 30, 7-day and 30-day clinical cure of CR PA BSI and identify mortality-related risk factors	MDR phenotype was associated with higher 30-day mortality (aHR 3.08, 95% CI 1.16–8.19; *p* = 0.024) No significant differences were found in clinical cure at 7 and 30 days
Rolo et al., 2022, [188]	BSI	Single center retrospective cohort study	Spain (328 episodes: MDR/XDR 114)	To investigate the impact of TTP of BC on 30-day mortality in PA BSI	A short TTP (≤ 16 h) was independently associated with increased 30-day mortality (41.0% vs. 19.5%, *p* < 0.001) The MDR/XDR phenotype (aOR 2.54, 95% CI 1.38–4.67; *p* = 0.002) was independently associated with 30-day mortality
Recio et al., 2021, [189],	BP	Single center retrospective cohort study	Spain (55 episodes: 32 XDR)	To assess the clinical and bacterial characteristics of patients with PA BP	XDR phenotype was associated with late mortality (aOR = 5.46, 95% CI 1.25–23.85; *p* = 0.024) and inappropriate empirical antimicrobial therapy (59.4% vs. 13.0%; *p* = 0.001)
Zhao et al., 2020, [187]	BSI	Single center retrospective cohort study	China (293 patients: CR 55, MDR 38)	To explore outcomes of acute leukemia patients with PA BSI and analyze risk factors of MDR and CR PA	MDR strains were associated with higher 30-day mortality (aOR 7.19, 95% CI 2.77–18.66; *p* < 0.001)
Recio et al., 2020, [42]	BSI	Single center retrospective cohort study	Spain (243 episodes: 87 XDR, 6 MDR)	To investigate prognostic factors affecting mortality in PA BSI, with attention to resistance and virulence	MDR phenotype was associated with inadequate empirical antimicrobial therapy (59.1% vs 20.0%, *p* < 0.001) and worse outcomes (early mortality 34.4% vs 11.3%, *p* < 0.001; late crude mortality 52.7% vs 21.3%, *p* < 0.001)
Zhang et al., 2020, [190]	BSI	Multicenter retrospective cohort study	China (215 patients: 40 CR, 41 MDR)	To assess the risk factors for in- hospital mortality and investigate the influence of resistance profiles on mortality	CR phenotype was associated with in-hospital mortality (aOR 4.48, 95% CI 1.08–18.57; *p* = 0.038) CR and MDR profiles were associated with 5-day mortality (Log-rank, *p* < 0.05)
Babich et al., 2020, [191]	BSI	Multicenter retrospective cohort study	Israel, Sweden, Spain, France, UK, Australia, Germany, Slovenia, and Greece (2,396 episodes: 335 MDR)	To determine risk factors for 30-day mortality in PA BSI	MDR phenotype was associated with increased 30-day mortality (aOR 1.52, 95% CI 1.15–2.1; *p* = 0.004) and inappropriate empirical therapy (69.9% vs 76.6%)
Micek et al., 2015, [214]	NP	Multicenter retrospective cohort study	USA, France, Germany, Italy and Spain (740 patients: 226 MDR)	To determine the risk factors for MDR strains and the association with hospital mortality	MDR phenotype was associated with hospital mortality (aHR1.39, 95% CI 1.05–1.83; *p* = 0.021) and confirmed by Cox model-adjusted survival curve analysis
Peña et al., 2015, [5]	BSI	Post hoc analysis of a prospective multicenter cohort study	Spain (593 patients: 168 MDR, 81 XDR)	To determine whether the TTSS genotype is a useful prognostic marker of PA bacteremia mortality and the association with resistance profiles	The* exoU* genotype was associated with increased risk of early mortality (aHR, 1.90 [95% CI, 1.15–3.14]; *p* = 0.01) Late mortality was associated with MDR profiles The* exoU* genotype was positively linked to the moderately resistant phenotype and negatively linked to the XDR phenotype
Jeong et al., 2014, [217]	BSI	Single center retrospective cohort study	Korea (242 patients: 63 CR)	To assess the risk factors for mortality and to examine the impact of virulence factors and resistance on outcomes in patients with CRPA BSI	A 1% increase in the biofilm-forming ability was associated with a 1.10 times higher risk of mortality A significant correlation was observed between the biofilm-forming ability and elastase activity and their APACHE II scores
Peña et al., 2012, [183]	BSI	Prospective multicenter cohort study	Spain (632 episodes: 145 CR)	To assess the impact of CR on mortality in PA BSI	Although there was no difference in mortality before day 5 after the onset of the bacteremia, there was a time-dependent relationship between CR phenotype and mortality at day 30, but that association was determined by the severity of comorbidities with higher Charlson index scores having a lower impact on mortality (aHR, 9.9, 95% CI, 3.3–29.4; *p* < 0.001 for a Charlson score of 0 and aHR 2.6, 95% CI 0.8–8.0; *p* = 0.1 for a Charlson score of 5)
Morata et al., 2012, [194]	BSI	Single center prospective cohort study	Spain (709 episodes: 127 MDR)	To assess the influence of appropriate empirical antibiotic therapy and MDR on mortality in patients with PA BSI	MDR phenotype had higher 30-day mortality than non-MDR (17.2% vs 32.3%; *p* = 0.0001) due to a higher rate of inappropriate empirical antibiotic therapy (62.2% vs 27%; *p* = 0.0001) and also, in almost 50% of MDR episodes, appropriate empirical therapy consisted of amikacin monotherapy
Joo et al., 2011, [193]	BSI	Single center retrospective cohort study	Republic of Korea (202 episodes: 42 MDR)	To identify predictors of mortality and evaluate the clinical impact of antimicrobial resistance (ceftazidime, piperacillin, imipenem, fluoroquinolones, aminoglycosides or MDR resistance) on outcomes	PA resistant to ceftazidime or imipenem was associated with higher mortality (aOR 3.35, 95% CI 1.38–8.10; *p* = 0.007 and aOR 2.74 95% CI 1.02–7.37; *p* = 0.046, respectively) Antimicrobial-resistant BSI had higher clinical failure rates regardless of the type of antimicrobial resistance (all *p* < 0.05) The 30-day mortality rate was higher, and duration of hospital stay was longer in the resistant group, with the exception of fluoroquinolone resistance and aminoglycoside resistance
Tumbarello et al., 2011, [6]	BSI	Multicenter retrospective case-case–control and cohort study	Italy (106 cases and 212 controls: 46 MDR)	To identify factors associated with in-hospital mortality (at 21 days) and compare survivors and non-survivors	MDR phenotype was associated with higher 21-day mortality (aOR 3.31, 95% CI 1.27–8.59; *p* = 0.01) The survival curve also showed that the MDR phenotype had a lower probability of survival than the non-MDR group
Tam et al., 2010, [192]	BSI	Single center retrospective cohort study	USA (109 episodes: 25 MDR)	To evaluate the impact of MDR PA BSI on outcome	MDR phenotype was associated *with* inappropriate empirical therapy (44.0% vs 6.0%, *p* < 0.001), longer prior hospital stays (32.6 ± 37.3 and 14.4 ± 43.6 days, *p* = 0.046), 30-day mortality (aOR 6.82; 95% CI 1.9–24.0; *p* = 0.003) and shorter time to mortality (*p* = 0.011)
Wei et al., 2023, [196]	BSI	Single center retrospective cohort study	China (198 episodes: 34 CR PA)	To evaluate risk factors for CR PA BSI and outcomes of PA BSI	CR phenotype was not associated with negative outcome (hospital mortality or clinical failure) (aOR 1.61, 95% CI 0.62–4.17;* p* = 0.32) Previous exposure to carbapenem (aOR 3.51, 95% CI 1.35–9.11; *p* = 0.010) was an independent risk factor for CR PA BSI
Montero et al., 2020,^197^	BSI	Single center retrospective cohort study	Spain (382 episodes: 122 XDR)	To evaluate the impact of XDRphenotype on mortality in PA BSI	XDR phenotype was not associated with 14 (aHR 1.07, 95% CI 0.68–1.67; *p* = 0.777) or 30-day mortality (aHR 1.14, 95% CI 0.77–1.69; *p* = 0.504)
Tumbarello et al., 2020, [215]	UTI	Multicenter retrospective case–control study	Italy (242 episodes: 65 MDR)	To characterize PA UTIs and identify risk factors for PA MDR infections	MDR was associated with clinical treatment failure at 72 h (aOR 2.67 95% CI 1.38–5.20; *p* = 0.001), but not at day 7 (23.1% vs 14.1%, *p* = 0.09) or with mortality (9.2% vs. 12.4%, *p* = 0.49) Median hospital stay for MDR cases was longer (48 vs. 22 days; *p* ≤ 0.001)
Denis et al., 2019, [211]	VAP	Multicenter retrospective cohort study	France (230 patients with 286 episodes: 73 MDR)	To evaluate the relationship between MDR PA profile and 30-day mortality in VAP	MDR phenotype was not associated with 30-day mortality (aHR 0.87, 95% CI 0.52–1.45; *p* = .59) The MDR group received more inadequate empiric antibiotic therapy (18.4% vs 4.7%; *p* = .002) and had more recurrence of VAP (second or third episodes) (*p* = 0.018)
Ferreiro et al., 2017, [216]	UTI	Single center retrospective cohort study	Spain (62 episodes: 13 MDR)	To evaluate mortality in PA UTI and the impact of antibiotic treatment on survival	MDR phenotype was not associated with 30-day mortality (23.5 vs 15.6%, *p* = 0.4)
Suarez et al., 2010, [182]	BSI	Single center retrospective cohort study	Spain (116 patients with 121 episodes: 33 CR- PA)	To determine the influence of CR PA BSI on attributable mortality (within 7 days from the onset of bacteremia and after excluding other causes of death)	CR phenotype was not associated with infection-attributable mortality (aOR 1.1 95% CI 0.6–2.1; *p* = 0.69) A Kaplan–Meier survival analysis showed that in the first 48 h after the onset of BSI, the cumulative mortality proportion was lower in the CR PA group (13% vs. 50%; *p* = 0.026)
Peña et al., 2013, [208]	VAP	Single center retrospective cohort study	Spain (83 patients with 91 episodes: 60 MDR and 42/60 XDR)	To determine the impact of resistance on VAP outcome (early and crude mortality)	Non-MDR PA was associated with adequate empirical (68% vs. 30%; *p* < 0.001), definitive antimicrobial therapy (96% vs. 50%; *p* < 0.001) with a trend towards early mortality (29% vs. 15%; *p* = 0.06), and with no association with crude mortality Multiorgan dysfunction syndrome was the only predictor of crude mortality (aOR 4.31, 95% CI 1.14–16.2; *p* = 0.03)
Tumbarello et al., 2013, [209]	VAP	Single center retrospective cohort study	Italy (110 episodes: 42 MDR)	To study the impact of MDR profile and inadequate initial antibiotic therapy on ICU mortality and duration of MV	MDR infections had significantly longer median periods of post-pneumonia onset MV (15 [12–18] days vs 10.5 [6.5–13] days; *p* = 0.01)
Planquette et al., 2012, [210]	VAP	Multicenter retrospective cohort study	France (314 patients with 393 episodes: 56 MDR)	To determine recurrence of PA VAP prognosis and identify associated risk factors	MDR phenotype was not associated with treatment failure (*p* = 0.4) but was linked to prolonged length of ICU stay (aHR 0.6, 95% CI 0.4–1.0; *p* = 0.048)
Kaminski et al., 2011, [212]	VAP	Multicenter prospective cohort study	France (361 patients with 223 episodes: 70 PR PA)	To estimate the impact of PR on outcomes in PA VAP (UCI mortality, hospital mortality and recurrence)	PR phenotype was not linked to increased UCI mortality (aOR 0.73, 95% CI 0.32–1.69; *p* = 0.46), hospital mortality (aOR = 0.87, 95% CI: 0.38–1.99; *p* = 0.74) or higher recurrence (*p* = 0.83) Adequate antimicrobial therapy was more frequently delayed in the PR group (*p* = 0.007)
Combes et al., 2006, [213]	VAP	Multicenter prospective randomized study comparing 8 vs 15 days of antibiotics	France (115 episodes: 64 PR PA)	To study the impact of PR on outcome in PA VAP for patients who had received appropriate empiric antibiotics	PR phenotype was not associated with 28-day mortality (aOR 2.00, 95% CI 0.72–5.61; *p* = 0.044), higher recurrence rates (*p* = 0.550), duration of mechanical ventilation (*p* = 0.842) or longer ICU stay (*p* = 0.394)
Carmeli et al., 1999, [199]	BSI	Single center retrospective cohort study	USA (489 episodes in 421 patients: 144 PA resistant to any of the study antibiotics (piperacillin, ceftazidime, ciprofloxacin, and imipenem))	To determinate the clinical and economic impact of antibiotic resistance in PA BSI	Emergence of resistance was associated with longer hospital stay (*p* < 0.01) Baseline resistance was not associated with mortality (aOR 1.3 95% CI 0.6–2.8; *p* = 0.52) or longer hospital stay (*p* = 0.72) Neither baseline resistance nor emergence of resistance was associated with hospital charge, but there was a trend toward increased total charges in patients with emergence of resistance (difference $7340, *p* = 0.14)

BC, blood cultures; BP, bacteremic pneumonia; BSI, bloodstream infections; CI, confidence interval; CR, carbapenem-resistant; DTR, difficult-to-treat resistance; aHR, adjusted hazard ratio; MDR, multidrug-resistant; MV, mechanical ventilation; NP, nosocomial pneumonia; aOR, adjusted odds ratio; PA, *Pseudomonas aeruginosa*; PR, piperacillin resistance; TTP, time-to-positivity; UTI, urinary tract infection; VAP, ventilator-associated pneumonia; XDR, extensively drug-resistant

Apart from relevant clinical variables and inappropriate empirical therapy, different studies have identified an association between antimicrobial resistance and worse clinical prognosis in bloodstream infections [6, 42, 183–193]. In other studies, however the MDR/DTR phenotype was not associated with poor prognosis in bloodstream infections [183, 196–199].

With respect to respiratory tract infections, most studies have analyzed outcomes in ventilator-associated pneumonia (VAP). Although some reported correlations between different resistant microorganisms other than *P. aeruginosa* and mortality [200–204], other studies observed worse clinical outcomes associated with MDR pathogens, such as longer intensive care unit (ICU) stay, prolonged duration of mechanical ventilation (MV) and higher rates of microbial persistence, but no impact on mortality [205–207]. Concerning MDR/DTR *P. aeruginosa* infections, associations with longer duration of MV, longer ICU stay and longer delay to appropriate antimicrobial therapy administration have also been noted, but with no significant differences in treatment failure and/or death [208–213].

In terms of UTI, few studies have analyzed infection outcomes according to *P. aeruginosa* resistance patterns. In two retrospective studies it was found that the MDR phenotype was not associated with in-hospital and all-cause late mortality [215, 216].

Regarding the association of virulence determinants and resistance profiles, not many studies have investigated its impact on clinical outcomes. In a multicenter study previously mentioned by Peña et al*.*, including 593 *P. aeruginosa* bloodstream infections, was investigated the interplay between the T3SS genotypes (*exoS*,* exoT*,* exoU* and* exoY* genes), the MDR phenotypes, and the influence in mortality. It was observed that *exoS* + isolates were more frequently MDR than *exoU* + isolates and *exoU* genotype was positively related to the moderately resistant phenotype and negatively linked to the XDR phenotype. They found that early mortality was associated with *exoU* + isolates and late mortality was not conditioned by the T3SS genotype but was independently associated with the MDR phenotype [5]. These results support that T3SS genotype is the most important virulence determinant in *P. aeruginosa* and *exoU* + is associated with worse clinical otucomes. Jeong et al. also analyzed, in a study conducted in Korea, the impact of virulence factors on clinical outcomes in 63 CR bacteremia strains. They found that the capacity of the strain to form biofilm was an independent predictive factor for mortality. In addition, the biofilm-forming ability and elastase activity of strains were correlated with the severity of the infection (determined by the Acute Physiology and Chronic Health Evaluation II (APACHE II) score) [217].

## Economic burden of multidrug resistance in *Pseudomonas aeruginosa* infections

Apart from their clinical impact and threat to patient health, P. *aeruginosa* infections and the interplay between resistance and virulence are associated with significant burden and cost, which are worth reviewing here. The additional cost of a single case of *P*. *aeruginosa * infection for example has been estimated to be approximately 18–19,000 Euros [218, 219].

Antimicrobial-resistant infections have been shown to be associated with a higher economic burden compared to those caused by susceptible strains. In a recent meta-analysis and systematic review of the economic cost of resistance (*P. aeruginosa* was among the three most studied bacteria), the attributable cost of resistant infection ranged from − US$2,371.4 to + US$29,289.1 per patient episode, the mean excess length of stay was 7.4 days, and the odds ratio of readmission for patients with resistant infection was 49.2% higher than for those with susceptible strains [220].

For patients with MDR *P. aeruginosa* nosocomial infections, the total mean economic cost per admission is higher than for those with non-resistant strains (15,265 vs. 4933 Euros) [221]. For respiratory infections, in a U.S. study, the excess cost per case was US$22,370, the average adjusted excess length of stay was 6.7 days and the adjusted readmission rate was 16.2% versus 11.1% in non MDR infections [222]. Carbapenem-resistant compared to carbapenem-susceptible *P. aeruginosa* infections are also associated with longer median length of stay, higher total hospital costs (median $6082.0 vs $4954.2) and daily hospital costs (median $236.1 vs $223.6) [223, 224]. Meanwhile, a recent retrospective multicenter study in China analyzing the clinical and economic burden of CR infection or colonization caused by *Klebsiella pneumoniae*, *P. aeruginosa* and *Acinetobacter baumannii* found that the CR phenotype was related to increased total hospital cost in all microorganisms ($4605 in CR *P. aeruginosa*) and excess length of stay (5.4 days in CR *P. aeruginosa*) [225].

Finally, it should be also noted the consequences associated with the high cost of the newly antimicrobials introduced in the market for difficult to treat infections or those expected to be approved in the future. In a recent study estimating the annual incremental costs of treating, among others, difficult-to-treat Gram-negative bacteria with new drugs in US hospitalized patients, was estimated that the cost of a 14-day course for MDR *P.aeruginosa* was doubled with new drugs; and the annual incremental costs of treating difficult-to-treat Gram-negative bacteria ranged from 30 million to over 500 million US$ [226]. This situation is especially worrisome in lower-middle and low-income countries where the clinical and economic impact of antimicrobial resistance is more pronounced [227, 228] and the access to healthcare facilities and new antimicrobials is limited [229].

Therefore, although the question of the clinical consequences of resistant *P. aeruginosa* infections*,* particularly the impact on mortality, remains open (with many different studies showing conflicting and controversial results, as shown above), the evidence that antibiotic-resistant infections have a much greater economic impact on healthcare systems is overwhelming. Further research is needed therefore to optimize antimicrobial use and control strategies for multidrug-resistant infections.

## Conclusions

Although it has been shown that MDR/DTR *P. aeruginosa* is associated with an increased economic burden, attributable to prolonged length of stay and higher average total costs, the topic of the biological implications of antibiotic resistance for virulence and fitness in *P. aeruginosa,* and thus for patient outcome, is currently debated. As we explained above, although it is generally assumed that acquisition of resistant determinants is associated with a fitness cost, several studies support that resistance mutations may not be associated with a decrease in virulence and/or that certain compensatory mutations may allow MDR/DTR strains to recover their initial fitness. Nonethless, mutations also can have environment effects by increasing or decreasing fitness under certain conditions, for example influenced by the antimicrobial treament or the host. Therefore, environmental variability may modify the tendency to compensatory mutations if the fitness of resistance mutations differs among settings.

For the clinical consequences of MDR/DTR infections, it is difficult to establish a direct association between MDR/DTR profile and mortality attributable to the infection. Conflicting results for the clinical outcomes of MDR/DTR *P. aeruginosa* infections have been reported, with different possible explanations for the inconsistent results, including treatment and host-dependent factors. Patients with MDR/DTR infections are at increased risk of receiving inappropriate empirical antibiotic therapy and alternative therapies (second-line treatments) that are less effective than those used for treating non-MDR infections. In addition, host-dependent factors associated with MDR/DTR infections may also partly explain the worse outcome in these patients, as these infections usually occur in patients with severe preexisting comorbidities, more severe illnesses and longer prior hospital stays. Certain methodological limitations, such as the retrospective study design or studies with small sample sizes may also act as biases. Consequently, further studies are needed to clarify the real effect of infections caused by MDR and DTR *P. aeruginosa.*

In terms of the basic knowledge reviewed here, a number of conclusions can be drawn. The first is that the interplay between resistance and virulence is highly complex, and is reflected in the remarkable occurrence of results that contradict each other for specific resistance mechanisms. Complexity is also reflected in the variability of costs depending on the mechanism: high burden vs innocuous vs enhancement of virulence. Second, from the information collected, it can also be gathered that, although a lot of data is available, there are several gaps in knowledge. To give some examples, the field of compensatory evolution associated with different resistance mechanisms appears not to be well studied in *P. aeruginosa*. Another gap that has not been filled is determination of the specific burden associated with most horizontally acquired β-lactamases, as well as of the mechanisms determining resistance to newly introduced drugs (ceftolozane/tazobactam, imipenem/relebactam, ceftazidime/avibactam and cefiderocol) or those likely to be approved in the near future (aztreonam/avibactam, cefepime/zidebactam or cefepime/taniborbactam) [230]. A holistic approach is required to understand all aspects of the balance between resistance and fitness/virulence in *P. aeruginosa*, including basic research and clinical perspectives. Deep knowledge of the topic is essential to find anti-virulence targets, and to understand the intricate landscape of the co-evolution of resistance and virulence in the context of acute infection. This could help to understand how individual antibiotic resistance mechanisms fine-tune *P. aeruginosa* virulence-related behavior thereby making it possible to predict modulation in the pathogenesis of infection and the consequences for the patient along over time, knowledge that can be exploited to good effect in clinical practice [231]. It is anticipated that opening up or digging deeper into the fields of research mentioned above will be of great help in combating one of the greatest challenges to public health in the twenty-first century, namely, *P. aeruginosa* resistance.

## Data Availability

No datasets were generated or analysed during the current study.
